# Mendelian randomization combined with multi-omics explores the relationship between heart failure and cancer

**DOI:** 10.7150/jca.94142

**Published:** 2024-03-25

**Authors:** Tian Sun, Na Mei, Yanting Su, Shigang Shan, Wenbin Qian, Mengxi Li, Zhenwang Zhang

**Affiliations:** 1 Hubei provincial key laboratory of diabetic cardiovascular diseases, Xianning Medical College, Hubei University of Science and Technology, Xianning 437100, Hubei, People's Republic of China.; 2 School of Basic Medical Sciences, Xianning Medical College, Hubei University of Science and Technology, Hubei University of Science and Technology, Xianning 437100, Hubei, People's Republic of China.; 3 School of Pharmacy, Xianning Medical College, Hubei University of Science and Technology, Xianning 437100, Hubei, People's Republic of China.; 4 School of Nuclear Technology and Chemistry & Biology, Hubei University of Science and Technology, Xianning 437100, Hubei, People's Republic of China.

**Keywords:** HF, cancer, MR, co-localisation analysis, SMR, transcriptomic analyses

## Abstract

**Background**: Whether there is an association between HF (HF) and cancer has not been conclusively established, and it is not clear whether patients with cancer can share similar hospitalization strategies and outcomes with patients with HF.

**Methods:** Genome-wide association summary statistics were performed using a two-sample Mendelian randomization (MR) method for HF patients and cancer patients from the GWAS directory, with co-localization and Summary Data-Based Mendelian Randomization (SMR) analyses to identify HF-associated genes, and transcriptomic analyses to analyze the roles of these genes in the clinical diagnosis and targeted therapies of multiple cancer types.

**Results:** Two-sample MR analysis showed that increased risk of HF was associated with decreased risk of cervical, brain, breast, colorectal, lung, and skin cancers, and co-localization combined with SMR analysis identified ABO and SURF1 as HF-associated genes, and transcriptomic analyses showed that ABO is a risk factor for HF and a protective factor against cancer, whereas SURF1 is a protective factor against HF and a protective factor against cancer.

**Conclusion:** There was no causal relationship between heart failure and cancers (Cervical, brain, breast, colorectal, lung and skin cancers) risk factors, however there was a trend toward a negative causal relationship between heart failure and cancers (Cervical, brain, breast, colorectal, lung and skin cancers) occurrence.

## 1. Introduction

The International Agency for Research on Cancer (IARC), a cancer agency of the World Health Organization, has released updated estimates of the global burden of cancer as of 1 February 2024. Around 20 million new cancer cases and 9.7 million deaths are expected in 2022. With approximately 53.5 million survivors within five years of cancer diagnosis, about one fifth of people will develop cancer in their lifetime, and about one-ninth of men and one-two-twelve of women will die from cancer. An increasing number of new cases of cancer are projected to surpass 35 million globally by 2050. In this battle without smoke, cancer has become one of the world's biggest health and lifespan "killers"^1^. The worldwide prevalence of HF is estimated to range between 1 and 2 percent 2. HF, often known as the "cardiovascular cancer," is a severe form of many cardiovascular diseases. It is characterized by high mortality and morbidity rates 3. According to the clinical consensus released by the European Society of Cardiology HF Association (ESC-HFA) in 2023, the in-hospital mortality rate for patients with HF and reduced ejection fraction (HFrEF) was recorded at 3.4%, while those with HF and mid-range ejection fraction (HFmrEF) had a mortality rate of 2.1%. Additionally, patients with HF and preserved ejection fraction (HfpEF) exhibited an in-hospital mortality rate of 2.2%. The respective annual hospitalization rates for these groups were reported as follows: HFrEF (48%), HFmrEF (35%), and HfpEF (42%). According to the American Heart Insufficiency Association, there are almost 6.7 million adults aged 20 and above in the United States who experience this condition during their lifetime 4. The China Cardiovascular Health and Disease Report 2020, released by the National Centre for Cardiovascular Disease, indicates that over 3.3 million individuals have been diagnosed with heart disease, while around 8.9 million individuals are suffering from severe insufficiency. The five-year mortality rate for HF exceeds that of many common malignancies, such as prostate cancer and bladder cancer, by up to 50%. Moreover, based on a survey carried out in six cities in China, the incidence of age-specific HF is 1.1 percent, impacting approximately 12.1 million urban-central persons aged 25 and above who experience cardiac insufficiency 5. While there has been an advancement in the uniformity of treatment for patients with HF, the rates of death and readmission to the hospital continue to be significant, resulting in substantial financial strain on the healthcare system.

Although cancer and HF are distinct medical conditions, they may occasionally manifest similar symptoms including but not limited to weight loss, depressive symptoms, appetite changes, and weakness 6. They also exhibit a number of additional risk factors in common, such as aging, obesity, smoking, hypertension, diabetes 7-9, and excessive alcohol consumption 10, genetic predisposition, compromised cardiopulmonary function, and drug toxicity 11. In conjunction with extended periods of inactivity, excessive tension, and prolonged sleep, these elements contribute to a heightened mortality rate and an increased financial strain on the healthcare system.

Researchers are employing medical epidemiological research methodologies to investigate the population distribution and influential variables of HF and cancer, aiming to elucidate the potential correlation between the two.An exemplary example is the Golestan cohort study, which was published in BMC Medicine and has a sample size exceeding 50,000 participants. This investigation confirmed the link between ABO blood type and the vulnerability to death associated to cardiovascular disease. Furthermore, it investigated, for the initial instance, the correlation between cancer and fatality. Studies have shown that people with blood types A and B have a higher likelihood of developing gastric cancer, while non-O blood types are closely linked to increased rates of overall mortality and mortality from cardiovascular disease 12, 13. Liang Chang and colleagues identified a notable association between blood type A and the incidence of malignant melanoma of the skin, in contrast to blood type O 14. These findings indicate a novel association between ABO blood type and the occurrence of cancer and heart disease. Moreover, accumulating evidence supports the substantial role of glomerular dysfunction in promoting the aging process, enhancing tumor growth, and enabling the transfer of cancer cells during transplantation 15, 16. Fibromyalgia has been identified as the main cause of the heart's inability to produce and utilize energy. This error can lead to a detrimental cycle of ventricular failure, defined by an imbalance of calcium, oxidative stress, damage caused by proteins, and death of heart muscle cells 17. Targeting fibromyalgia directly may potentially be an effective treatment for HF 18. SURF1 functions as a specific co-factor for phosphoric c oxidase, a protein that impedes the formation of conventional COX molecules in phosphates, hence impacting energy production. Regrettably, the current corpus of research pertaining to the correlation between SURF1 and both cancers and HF is inadequate. Therefore, additional examination and scrutiny are necessary.

**Figure 1:**
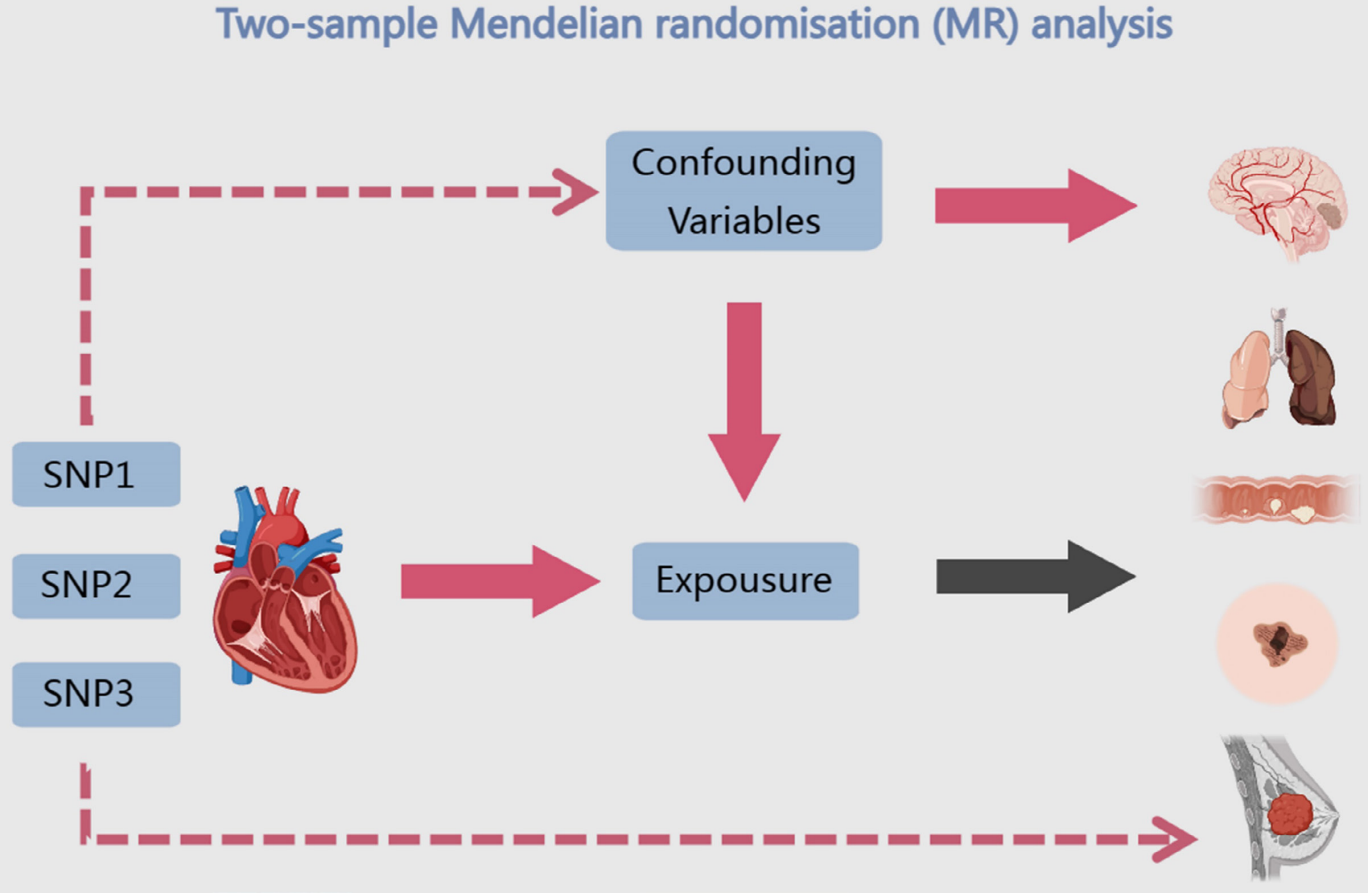
The investigation's design is depicted using a flow diagram.

Due to numerous factors, including study methodology, participant count, follow-up duration, geographic heterogeneity, demographic characteristics, and the specific types of cancer under investigation, the association between HF and the risk of developing cancer remains a topic of ongoing controversy 19. Several studies have shown that HF is associated with an increased risk of malignancy 20-23. Nevertheless, Irene Fernández-Ruiz and colleagues, after analyzing numerous factors, demonstrated that HF is not associated with general cancer, localized cancer or cancer-specific mortality 24, 25. Notable advancements have been achieved in cancer and HF research in the past few decades. Nevertheless, the existing research is constrained by the drawbacks of observational studies, thus it can solely establish a correlation between HF and a heightened susceptibility to cancer. Consequently, it is not feasible to definitively elucidate whether there exists a direct causal and biological connection between the two ailments. MR is an epidemiological research method that utilizes genetic information to assess causal relationships between variables. By using genetic variation as an instrumental variable, MR avoids confounding factors and provides reliable causal relationships. Figure 1 depicts the study design, which employs a double sample MR approach to gather genetic variation data from extensive genomic databases, such as the People's Genome Programme and the British Biological Bank. The main objective of this study is to investigate the relationship between cancer and hyperlipidemia through co-localization analysis, SMR analysis and transcriptional analysis. The results are expected to deepen our understanding of the relationship between hyperlipidemia and cancer, reveal common risk factors, lay the foundation for developing preventive measures, and provide direction for the use of magnetic resonance technology in disease investigation.

## 2. Methods

### 2.1 Two-sample MR analyses

Our method of choice for examining the causal link between cancer and HF was a two-sample MR analysis. In this analysis, single nucleotide polymorphisms (SNPs) were employed as instrumental factors, cancer was the outcome variable, and HF was the exposure variable of interest. Based on the following hypotheses, two-sample MR analyses were performed: (I) the instrumental variables were highly correlated with the risk of HF; (II) the instrumental variables only increased the risk of cancer by influencing the risk of HF; and (III) the instrumental variables were unaffected by confounders. Genome-wide association summary information for 47,309 HF patients and 37,976 cancer patients (all European patients) was gathered from the GWAS catalog (https://www.ebi.ac.uk/gwas/). Summary association statistics for the exposure factors were derived using HF-specific GWAS data (ebi-a-GCST00009541), which included 47,309 HF patients and 93,0014 controls with a total of 7,773,021 SNPs (Table 1). Pooled association statistics for six different cancer types-cervical, brain, breast, colorectal, lung, and skin cancers-were included in the cancer-specific GWAS data and were utilized as pooled association statistics for the outcome variable. We used a range of MR techniques, including Egger regression, weighted median, inverse variance weighting (IVW), basic and weighted models, to conduct robust assessments of causation. The criterion of a *P* value <0.05 produced by at least one MR method was used to establish the significance of the etiological influence of HF on cancer. We adopted a criterion of *P*<1×10^-8^ to identify genetic variations linked to the risk of HF, based on prior research.

### 2.2 SMR analysis based on pooled data

The SMR approach serves as a robust mechanism for consolidating findings from Genome-Wide Association Studies (GWAS) and Expression Quantitative Trait Loci (eQTL) inquiries. Its primary function is to evaluate the multifaceted relationships inherent in the interplay between gene expression levels and the complex traits that are the subject of study. By utilizing this method, researchers can investigate whether SNPs exert their influence on phenotypes through the mediation of gene expression. Gene therapy targets for a wide range of complicated disorders, including diabetes and coronary heart disease, have been identified using this technique on a large scale. The SMR database (https://yanglab.westlake.edu.cn/) MR provided the "# V8 release of the GTEx eQTL/sQTL summary data" that were used in this investigation.

### 2.3 MCA

MCA is a method used to investigate the interrelationships and mechanisms of several genetic variants or biomarkers involved in the progression of a shared disease. This analysis involves examining the MCA of these variants or biomarkers to get insights into their potential interactions and contributions to the disease. A comprehensive collection of information on all eQTL snp, encompassing both cis and trans snp, was achieved by integrating eQTL data and GWAS data derived from multiple tissues. When the co-localization of the GWAS signaling pathway with an eQTL is observed, it suggests that the GWAS locus may influence the expression phenotype of the target gene. The identification of the target gene at the HF risk locus is determined by the Log Bayes Factor (LBF) value. A higher LBF value indicates that the target gene is more likely to be associated with the risk locus for HF. In instances when a larger LBF is observed, it indicates a more robust correlation between the locus and the gene. In this study, we utilized GWAS data pertaining to HF, specifically the dataset labeled "ebi-a-GCST00009541." Additionally, we incorporated eQTL data from the Genotype-Tissue Expression (GTEx) project, which encompasses whole blood and whole brain samples. Specifically, we focused on GTEx data that pertained to blood and cardiac tissues, encompassing myocardial tissues, atria, ventricles, atrioventricular node, sinus node, and heart valves. The GTEx project can be accessed at https://www.gtexportal.org/home/index.html. Co-localization analysis was conducted to assess the risk of HF using the coloc R program, specifically focusing on significant MR findings.

### 2.4 Transcriptomics analysis

We conducted an analysis of the expression profiles of cancer risk genes using transcriptomics data obtained from The Cancer Genome Atlas (TCGA) database, specifically utilizing RSEM-standardized RNASeq gene expression profiles. The TCGA database can be accessed at https://gdc.cancer.gov/.

## 3. Results

### 3.1 MR Analysis Shows Inverse Risk Trend, But No Causal Relationship, Between Hypertension and Cancer

Two-sample MR analysis were conducted using SNPs as instrumental variables. The exposure of interest was HF, whereas the outcome was cancer. Summary association statistics for the exposure were derived from GWAS data pertaining to HF, whereas summary association statistics for the result were obtained from GWAS data specific to each cancer type.

Cervical cancer is a malignancy that arises in the vaginal portion of the uterus and the cervical canal in females. It ranks among the most prevalent malignant tumors affecting women, occupying the second position in terms of incidence among female malignancies. Globally, approximately 400,000 women receive a diagnosis of invasive cervical carcinoma annually, with an estimated 200,000 succumbing to the disease 26. The dataset for the GWAS pertaining to cervical cancer comprised a total of 563 individuals diagnosed with cancer and 198,523 individuals serving as controls. This dataset encompassed 9,822,229 SNPs, as indicated in Table 1. The findings from Mendelian randomization analyses showed a possible negative effect of HF risk on cervical cancer risk (*P*_MR-Egger_=0.145, *P*_weighted-median_=0.415, *P*_IVW_=0.367, *P*_simple-mode_=0.87 and *P*_weighted-mode_=0.244) (Table S1), and assessment of heterogeneity showed little evidence of correlation (*Q*_MR-Egger_=1.01 and *P*=0.985,* Q*_IVW_=5.21 and *P*=0.634) (Table S1). Furthermore, the results of the horizontal pleiotropy studies provided limited support for the notion that pleiotropy was linked to this particular connection (*P*=0.086) (Table S1). Brain cancer, referred to as intracranial tumors in medical terminology, is characterized by the growth of neoplastic organisms within the cranial cavity. These tumors can originate from various sources such as the brain, meninges, nerves, blood vessels, and cerebral appendages. Additionally, they can also be formed through the metastasis of malignant tumors from other tissues or organs in the body to the cranium. The prolonged presence of brain cancer can lead to several detrimental effects including increased intracranial pressure, displacement of brain tissues, visual impairments, paralysis, psychiatric disorders, cerebral hernia, cerebral hemorrhage, and potentially even death 27. The 8,629,116 SNPs covered by the 606 cancer patients and 372,061 controls in the GWAS data for brain cancer are shown in Table 1. Mendelian randomization analyses suggest that HF risk may have a negative effect on brain cancer risk (*P*_MR-Egger_=0.461 *P*_weighted-median_=0.063, *P*_IVW_=0.069, *P*_simple-mode_=0.186 and *P*_weighted-mode_=0.172) (Table S1), with assessment of heterogeneity showing little evidence of correlation (*Q*_MR-Egger_=3.74 and *P*=0.711,* Q_IVW_*=3.80 and *P*=0.802) (Table S1). Moreover, there was minimal indication that pleiotropy was connected to the relationship in horizontal pleiotropy studies (*P*=0.816) (Table S1). Breast cancer can occur in men as well as women and is a malignant tumor originating from the mammary epithelium or ductal epithelium, primarily due to abnormal mammary cell growth, and it is one of the most heterogeneous and frequently diagnosed cancers among women worldwide 28. The breast cancer GWAS data includes 15,748 cancer patients and 18,084 controls, with 13,011,123 SNPs included (Table 1). Mendelian randomization analyses suggest that HF risk may have a negative effect on breast cancer risk (*P*_MR-Egger_=0.467, *P*_weighted-median_=0.607, *P*_IVW_=0.605, *P*_simple-mode_=0.699 and *P*_weighted-mode_=0.528) (Table S1). The study of heterogeneity revealed no indication of association (*Q*_MR-Egger_=72.5 and *P*=0, *Q*_IVW_=83.1 and *P*=0) (Table S1). Furthermore, horizontal pleiotropy tests revealed no indication of pleiotropy being related to this connection (*P*=0.346; Table S1). Colorectal cancer, which accounts for 11% of all cancer diagnoses, is a malignant tumor that begins in the mucosal epithelium or glands of the large intestine. The colorectal cancer GWAS data contained 5,657 cancer patients and 372,016 controls, totaling 11,738,639 SNPs (Table 1). Mendelian randomization analyses suggest that HF risk may have a negative effect on colorectal cancer risk (*P*_MR-Egger_=0.806,* P*_weighted-median_=0.839, *P*_IVW_=0.234, *P*_simple-mode_=0.933, and* P*_weighted-mode_=0.887) (Table S1). The study of heterogeneity revealed no indication of association (*Q*_MR-Egger_=4.27 and *P*=0.748, *Q*_IVW_=4.78 and *P*=0.781) (Table S1). The instrumental variables were not horizontally polytomous, according to horizontal polytomous analysis (*P*=0.5) (Table S1). Lung cancer is a neoplastic growth that manifests as a malignancy in the pulmonary region, originating from either alveolar or respiratory epithelial cells. On a global scale, lung cancer holds the highest prevalence and fatality rates among malignant tumors 29. 11,078,115 SNPs were covered by 2,671 cancer patients and 372,016 controls in lung cancer (ieu-b-4954) (Table 1). Mendelian randomization analyses suggest that HF risk may have a negative effect on lung cancer risk (*P*_MR-Egger_=0.116, *P*_weighted-median_=0.322, *P*_IVW_=0.251, *P*_simple-mode_=0.207 and *P*_weighted-mode_=0.308) (Table S1), with heterogeneity. The assessment showed little evidence of correlation (*Q*_MR-Egger_=4.02 and *P*=0,*Q*_IVW_=7 and *P*=0.778) (Table S1), whereas horizontal multivariate analyses showed the presence of heterogeneity, with instrumental variables possibly influencing the occurrence of the outcome through factors other than exposure factors (*P*=0.049) (Table S1). Skin cancer, which predominantly develops in sun-exposed regions of the skin, is a malignant neoplasm of skin cells. Its mortality and morbidity rates are progressively rising, rendering it a significant global public health concern 30. Skin cancer (ukb-d-C_SKIN) included 16,531 cancer patients and 344,663 controls covering 13,586,589 SNPs (Table 1). Mendelian randomization analyses suggest that HF risk may have a negative effect on skin cancer risk (*P*_MR-Egger_=0.492, *P*_weighted-median_=0.631, *P*_IVW_=0.778, *P*_simple-mode_=0.500 and *P*_weighted-mode_=0.589) (Table S1). Heterogeneity assessment showed little evidence of correlation (*Q*_MR-Egger_=7.92 and *P*=0.34,* Q*_IVW_=8.79 and *P*=0.36) (Table S1). In addition, horizontal pleiotropy analyses showed little evidence that pleiotropy was associated with this association (*P*=0.41) (Table S1).

In summary, when HF was used as the exposure factor of interest and cancer was the outcome, Mendelian randomization analyses did not reveal a significant causal effect of HF on lung, cervical, breast, brain, colorectal, or skin cancers, but there was a trend toward a reduced risk of lung, cervical, breast, brain, colorectal, or skin cancers from HF.

### 3.2 Co-localisation and SMR identify HF target genes

**Figure 2:**
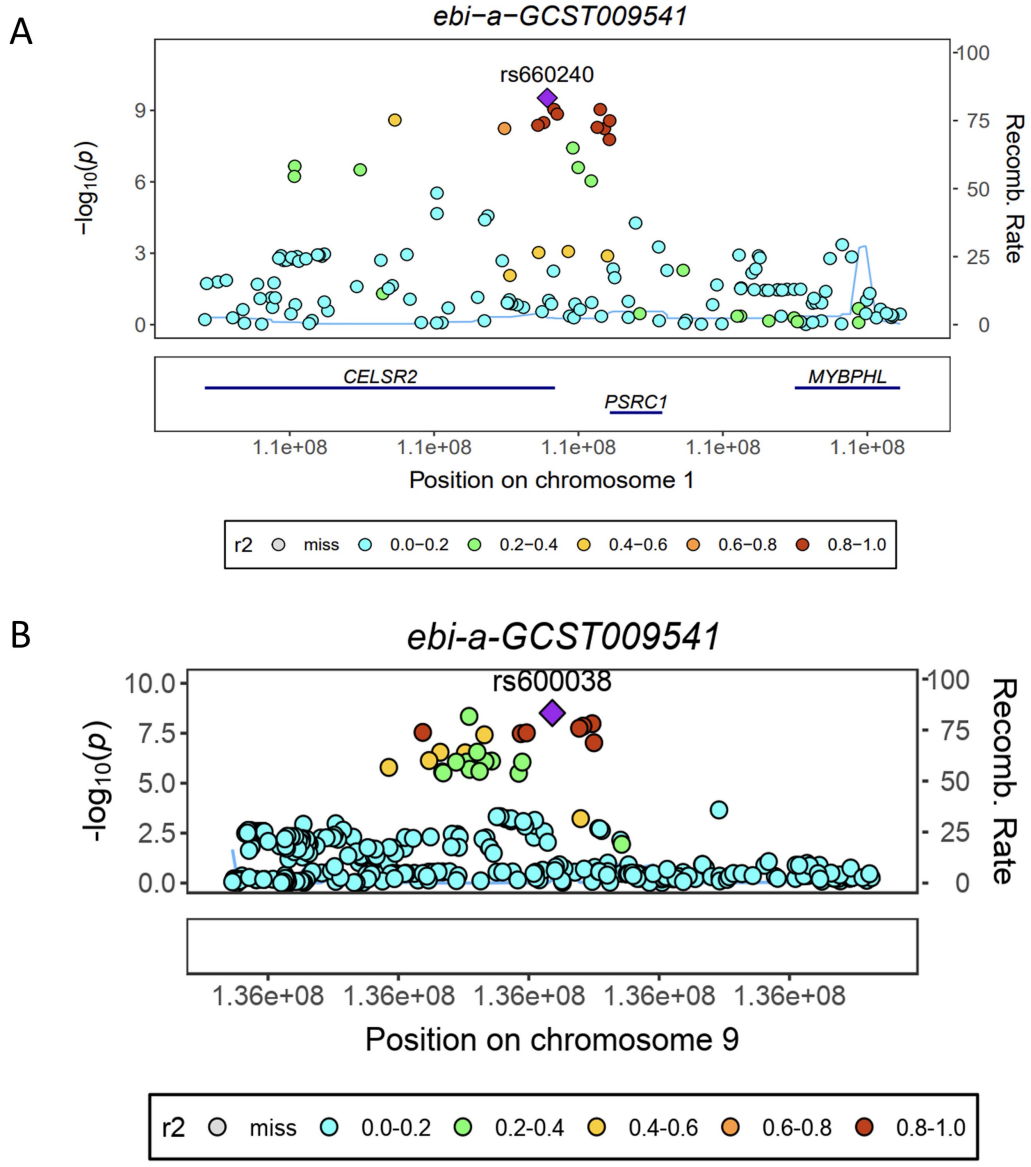
** Analysis of co-localization in HF.** (A) HF is impacted by rs660240's co-localization. (B) HF is impacted by rs600038's co-localization.

**Figure 3:**
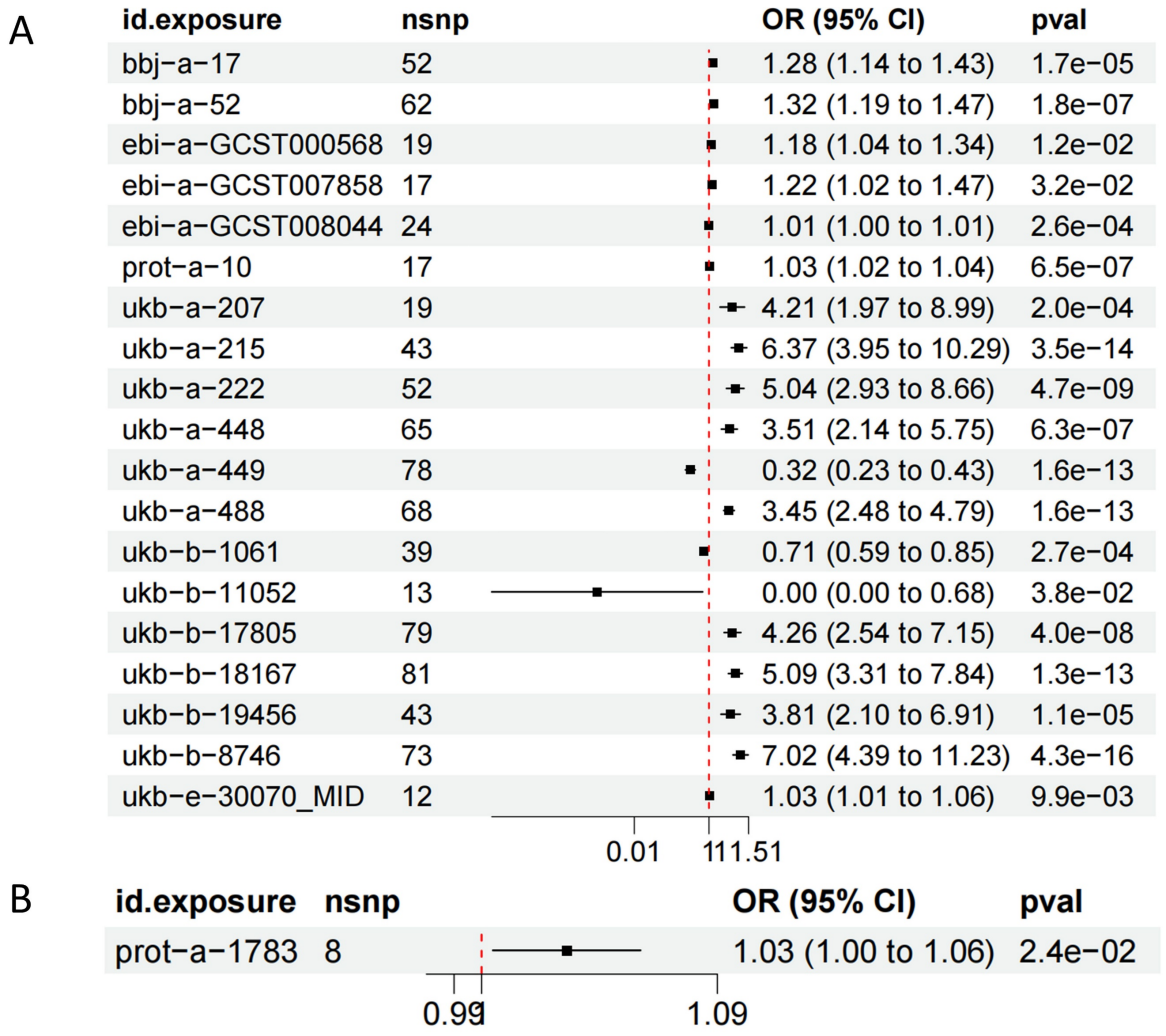
** Analysis of Mendelian randomized histology.** (A) Serological Mendelian randomization. (B) Mitochondrial Mendelian randomization.

**Figure 4:**
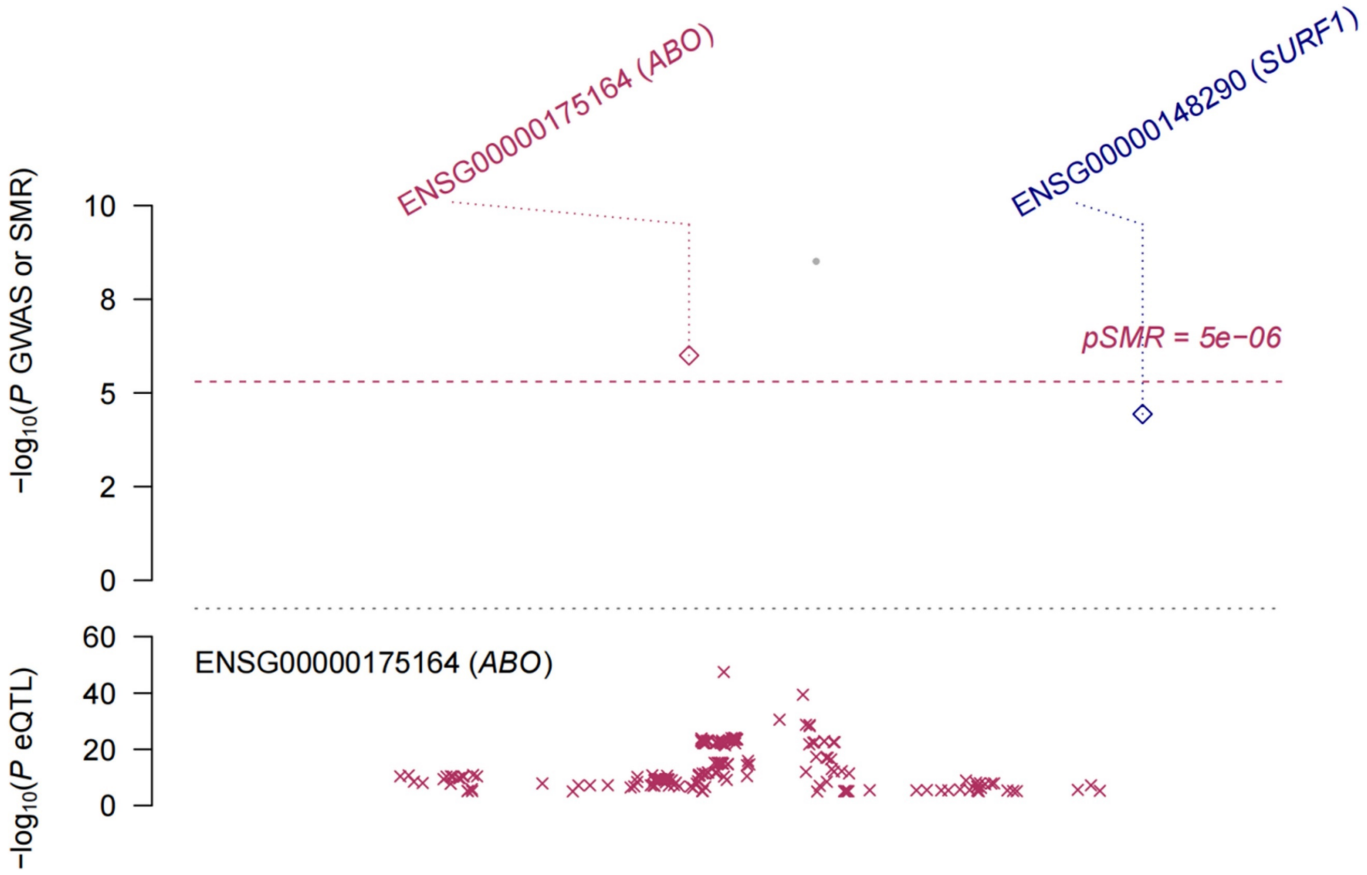
** The Mendelian randomization method, also known as SMR analysis, is employed in this study using pooled data.** There is a positive correlation between increased levels of ABO expression and an elevated vulnerability to HF. Conversely, heightened levels of SURF1 gene expression have been discovered to be linked with a reduced risk of HF.

eQTL refer to genetic variations that are linked to phenotypic traits related to gene expression. In order to investigate the potential sharing of causal genetic variation between SNPs associated with HF and eQTLs, only eQTL data specifically related to HF were utilized for co-localization analysis. This analysis revealed that the occurrence of co-localization effects involving rs660240 (Figure 2A) and rs600038 (Figure 2B) significantly influenced the manifestation of HF. In the analysis of drug target genes using MR, cis-expression quantitative trait loci (cis eQTL) situated within the genomic region of drug target genes are frequently employed as proxy variables for drug target genes to exert an influence on gene expression. Subsequent investigation through co-localization analysis has revealed that a SNP identified as rs600038 has the potential to impact the ABO blood grouping system on chromosome 9 via multiple molecular pathways (Table S2). The serological Mendelian randomization study conducted for HF revealed an association with 568 SNPs, as depicted in Figure 3A. Similarly, the mitochondrial Mendelian randomization analysis for HF demonstrated an association with 8 SNPs (Figure 3B). Through the utilization of SMR analysis, the investigation identified ABO and SURF1 as genes that are associated with HF. Notably, it was observed that the effect values of ABO and SURF1 exhibited opposing directions in relation to HF (Figure 4). Additionally, Increased ABO expression was associated with an increased risk of HF (*b_*_SMR_ > 0, *P_*_HEIDI_ < 0.05) (Table S3). Conversely, higher levels of SURF1 gene expression were associated with a decreased risk of HF (*b_*_SMR_ > 0, *P_*_HEIDI_ < 0.05). These findings collectively contribute to the understanding of the initiation and progression of HF.

### 3.3 Transcriptomic analysis

**Figure 5:**
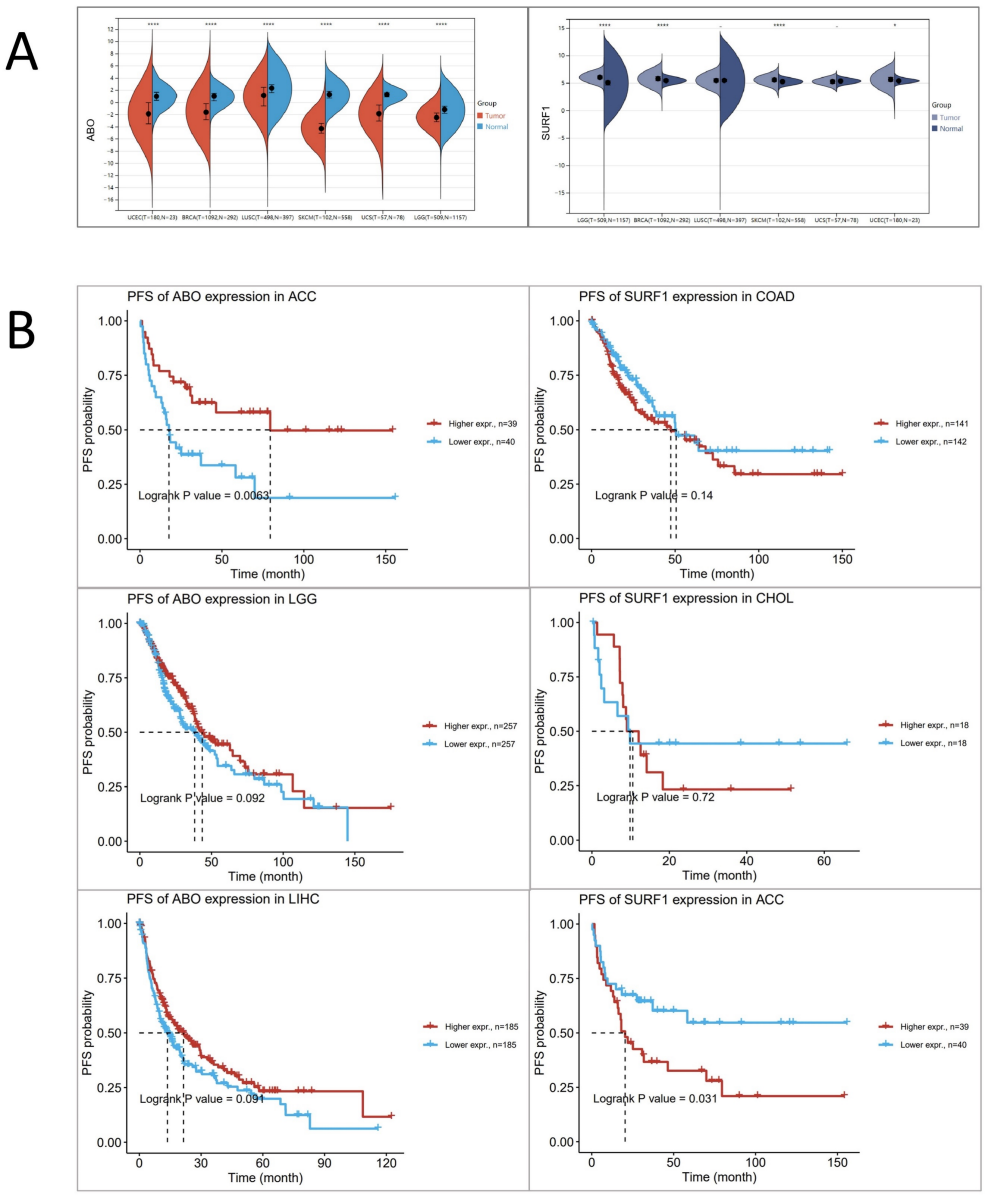
** Analysing the expression profile of risk genes in cancer based on transcriptomics data.** (A) Differential expression of ABO and SURF1 in normal and tumour samples. (B) Progression-Free Survival of ABO and SURF1.

**Figure 6:**
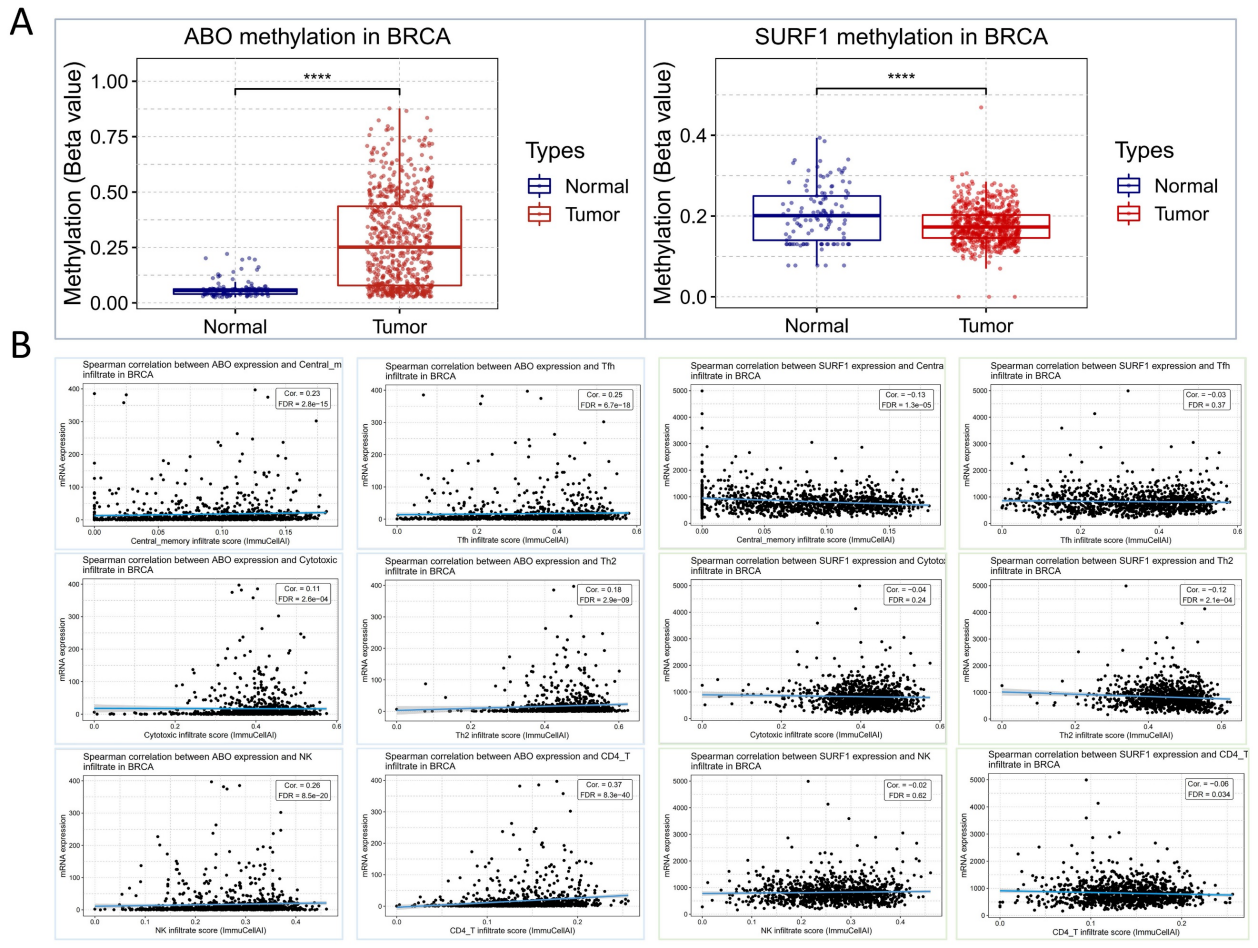
** Methylation of ABO and SURF1 in breast cancer and immunoexpression analysis.** (A) The methylation levels of ABO were found to be higher in individuals with BRCA compared to those in the normal group. Conversely, the methylation levels of SURF1 were observed to be lower in individuals with BRCA compared to those in the normal group. (B) There was a favorable link observed between the ABO blood group and the activation of various immune cell types, including Cytotoxic, Th2, Central memory, Tfh, NK, and CD4_T cells, in individuals with BRCA. In contrast, the SURF1 gene exhibited an inverse relationship with the immune cell activation patterns previously identified in breast cancer (BRCA).

Based on the aforementioned research findings, which demonstrate a positive association between elevated levels of ABO and the susceptibility to HF, as well as a negative association between elevated levels of SURF1 and the risk of HF, we sought to explore potential associations between HF and trends in cancer risk reduction. This was accomplished by employing transcriptomics techniques to examine the functions of ABO and SURF1 in cancer. The transcriptome profiles of many tumor forms were examined, including uterine corpus endometrial carcinoma (UCEC), brain lower grade glioma (LGG), breast invasive carcinoma (BRCA), lung squamous cell carcinoma (LUSC), uterine Carcinosarcoma (UCS), and skin cutaneous melanoma (SKCM). The statistical significance of the changes in gene expression between tumor and normal samples was then assessed using unpaired Wilcoxon Rank Sum and Signed Rank Tests. The findings from the analyses of significance of difference indicate that the expression of ABO gene was considerably decreased in cases of LGG, UCEC, BRCA, LUSC, SKCM, and UCS (Figure 5A). Conversely, the expression of SURF1 gene was dramatically increased in cases of LGG, UCEC, BRCA, and SKCM. Moreover, the analysis of survival data demonstrated that elevated SURF1 expression is linked to a worse prognosis in cases with adrenocortical carcinoma (ACC). In contrast, it has been observed that low ABO expression is linked to an unfavorable prognosis in ACC (Figure 5B). A further analysis was conducted to compare the roles of SURF1 and ABO in BRCA. The results indicated that methylation levels of ABO were significantly higher in the BRCA group compared to the control group. Conversely, methylation levels of SURF1 were found to be lower in the BRCA group compared to the control group (Figure 6A). The immunoassay data indicates a favorable association between ABO and BRCA-induced activation of many immune cell types, such as Th2, Central memory, Tfh, NK, and CD4_T cells. In contrast, a negative connection was discovered between the expression of SURF1 and the activation of immune cells (Figure 6B). The results indicate that SURF1 may have a pro-oncogenic role in cancer, whereas ABO may have oncogenic properties.

## 4. Discussion

Cancer ranks as the second most prevalent cause of mortality globally, behind cardiovascular disease, and contributes to around 21% of all fatalities 31, 32. In light of the shared metabolic pathways and clinical triggers between cancer and HF, a number of retrospective cohort studies, prospective cohort studies, and meta-analyses conducted in the past decade have consistently demonstrated an elevated risk of cancer and a poorer prognosis among individuals with HF, relative to the general population 33. These findings suggest that HF may potentially serve as a carcinogenic agent and a risk factor 34, 35. Even while cancer is notably more common in those with HF, the connection between HF and cancer is still up for discussion.

Based on a two-sample Mendelian randomization analysis, our results suggest that there may be a negative association between HF and a patient's susceptibility to multiple diseases, such as skin, breast, lung, brain, cervical, and colorectal cancers, but no statistically significant association was observed.

To enhance comprehension of the biological connection between the two, drug-targeting co-localization analysis and serological analysis of HF were employed. The co-localization of rs660240 and rs600038 was found to impact HF, and serological analysis of HF revealed that the SNP marker rs600038 could influence ABO gene expression via diverse protein levels. These findings were consistent with a prior investigation conducted by Hilde E. Groot et al. in the Netherlands, which determined that the ABO blood group was linked to eleven health and disease outcomes, the majority of which concerned cardiovascular conditions. The combined risk of HF was 10% greater among those with blood types A and B than among those with blood type O 36. Furthermore, SMR analyses revealed that SURF1, apart from ABO, is also a gene associated with HF, and that increased expression of this gene was correlated with a decreased risk of developing HF. SURF1, a mitochondrial protein predominantly present in alveolar type II epithelial cells, has been identified to influence redox balance, electron transport, and the assembly and functionality of the mitochondrial respiratory chain complex, among other processes^37^, while SURF1 does not have a direct impact on HF, its involvement in maintaining mitochondrial function, managing oxidative stress, regulating apoptosis, and facilitating mitochondrial autophagy contribute significantly to the development of HF. Additionally, studies have demonstrated a correlation between genetic variations in SURF1 and the occurrence of HF, pulmonary hypertension, and other related conditions 38.

Additional investigation into the functions of ABO and SURF1 in cancer has yielded noteworthy findings. Specifically, a considerable decrease in ABO expression has been observed in cases of LGG, UCEC, BRCA, LUSC, SKCM, and UCS. Conversely, there has been a significant increase in SURF1 expression in instances of LGG, UCEC, BRCA, and SKCM. Moreover, it has been observed that reduced ABO expression tends to be indicative of an unfavorable prognosis, while elevated SURF1 expression is associated with a poorer prognosis. Both hypomethylation events and hypermethylation events can lead to the occurrence of cancer. Hypomethylation affects the overall stability of the genome and activates proto-oncogenes, while hypermethylation leads to the silencing of tumor suppressor genes. This methylation phenomenon has been observed in the early stages of tumor development, even in populations susceptible to tumors 39. Elevated methylation levels of ABO were discovered in individuals with BRCA, relative to the control group, suggesting a potential involvement of ABO as an oncogene in cancer development. Conversely, reduced methylation levels of SURF1 were detected in BRCA patients compared to the normal group, indicating a plausible function of SURF1 as an oncogene promoter in cancer. Tumor-derived exosomes have been observed to interact with various immune effector cells, such as macrophages, monocytes, T cells, natural killer (NK) cells, dendritic cells (DCs), γδ T lymphocytes, regulatory T cells (Treg), myeloid-derived suppressor cells (MDSCs), mast cells, and B cells, within the hypoxic tumor microenvironment (TME).

These interactions contribute to the establishment of an immunosuppressive milieu within the tumor, facilitated by the release of factors that modulate the recruitment, phenotypes, and functions of these immune cells. The tumor microenvironment is characterized by an immunosuppressive milieu 40, and the immunoassay results indicated that ABO is linked to cytotoxicity, helper T-cell 2 (Th2), central memory, follicular helper T-cells (Tfh), natural killer (NK) cells, and CD4+ T-cells in BRCA. Additionally, SURF1 exhibited a positive correlation with the activation of cytotoxic, Th2, central memory, Tfh, NK cells, and CD4+ T-cells in BRCA. These findings suggest that the distinct expressions of ABO and SURF1 in cancer might be associated with the immune evasion mechanisms of tumors.

Based on the aforementioned research findings, we propose a bold speculation that clinical evidence suggests a potential correlation between HF and accelerated tumor growth. The long-term development of tumors during HF treatment may be influenced by underlying diseases, immune system disorders, or common risk factors such as smoking, obesity, and hypertension. Additionally, lifestyle changes during treatment or clinical drug toxicity, such as the cardiotoxicity of chemotherapy drugs like anthracyclines 41, indirectly contribute to an increased risk of HF and cancer. However, it is important to note that HF itself does not elevate the risk of developing cancer.

## 5. Conclusion and prospect

In summary, the results of our study, which incorporate epidemiological and transcriptome analysis of genetic data, suggest that there is no significant association between heart failure and an increased risk of cancer development. Nevertheless, heart failure negatively modulates the incidence of cancer. The molecular interconnections between heart failure and cancer risk may be mediated by ABO and SURF1, whilst the inverse relationship between heart failure and cancer risk may be influenced by methylation and tumor immune escape pathways.

The utilization of genetic information in epidemiological research has the potential to mitigate the inherent biases present in conventional observational studies. This approach holds promise for investigating the relationship between ABO blood group and mitochondrial function in HF, as well as for advancing the screening, diagnosis, and treatment of HF patients with concurrent cancer diagnoses in clinical settings. Nevertheless, it is crucial to acknowledge that a significant disparity persists between genetic research and its practical implementation in clinical settings. In order to elucidate the probable underlying mechanisms linking heart failure and cancer, as well as to offer novel insights and approaches for their diagnosis, treatment, and prevention, more functional research and clinical validation of this work are needed.

## Supplementary Material

Supplementary tables.

## Figures and Tables

**Table 1:** A summary of the data for Mendelian randomization analysis

id	trait	nsnp	ncase	sample_size	ncontrol	population	year
ebi-a-GCST009541	HF	7773021	47309	977323	930014	European	2020
ieu-b-4954	Lung cancer	11078115	2671	374687	372016	European	2021
ieu-b-4965	Colorectal cancer	11738639	5657	377673	372016	European	2021
ieu-b-4876	Cervical cancer	8506261	563	199086	198523	European	2021
ieu-a-1168	Breast cancer (GWAS)	13011123	15748	33832	18084	European	2015
ukb-d-C_SKIN	Cancer of skin	13586589	16531	361194	344663	European	2018
ieu-b-4875	Brain cancer	8629116	606	372622	372016	European	2021

## Abbreviations

HF: HF
MR: Mendelian randomization
SMR: Summary Data-Based Mendelian Randomization
MCA: Mendelian randomized colocalization analysis
GWAS: genome-wide association study
IARC: the International Agency for Research on Cancer
ESC-HFA: the European Society of Cardiology HF Association
HFrEF: HF with reduced ejection fraction
HFmrEF: HF with mid-range ejection fraction
HfpEF: HF with preserved ejection fraction
IV: instrumental variable
IVW: inverse-variance weighted method
SNPs: single nucleotide polymorphisms
eQTL: expression quantitative trait loci
LBF: Log Bayes Factor
GTEx: Genotype-Tissue Expression
ACC: Adrenocortical carcinoma
BRCA: Breast invasive carcinoma
CHOL: Cholangiocarcinoma
COAD: Colon adenocarcinoma
LGG: Brain Lower Grade Glioma
LIHC: Liver hepatocellular carcinoma
LUSC: Lung squamous cell carcinoma
SKCM: Skin Cutaneous Melanoma
UCEC: Uterine Corpus Endometrial Carcinoma
UCS: Uterine Carcinosarcoma
