# Supplementary material for: Mendelian randomization combined with multi-omics explores the relationship between heart failure and cancer
Source: J Cancer. 2024 Mar 25;15(10):2928–39. doi: 10.7150/jca.94142 (PMC11064263; doi:10.7150/jca.94142)
Supplement: Supplementary file 1 — Supplementary tables. [file jcav15p2928s1.pdf]

Table S1. Two-sample MR results of HF as the exposure and cancer as the outcome

Cervical cancer || id:ieu-b-4876	Method	nsnp	β	se	pval
Results from two sample MR	MR Egger	8	0.006	0.004	0.145
	Weighted median	8	-0.001	0.001	0.415
	IVW	8	-0.001	0.001	0.367
	Simple mode	8	-0.003	0.003	0.287
	Weighted mode	8	-0.003	0.003	0.244
	**Method**	**Q**	**Q_df**	**Q_pval**	
Heterogeneity tests	MR Egger	1.01	6	0.985	
	IVW	5.21	7	0.634	
	**egger_intercept**	**se**	**pval**		
Test for directional horizontal pleiotropy	0	0	0.086		
**brain cancer || id:ieu-b-4875**	**Method**	**nsnp**	**β**	**se**	**pval**
Results from two sample MR	MR Egger	8	-0.002	0.002	0.461
	Weighted median	8	-0.002	0.001	0.063
	IVW	8	-0.001	0.001	0.069
	Simple mode	8	-0.002	0.001	0.186
	Weighted mode	8	-0.002	0.001	0.172
	**Method**	**Q**	**Q_df**	**Q_pval**	
Heterogeneity tests	MR Egger	3.74	6	0.711	
	IVW	3.80	7	0.802	
	**egger_intercept**	**se**	**pval**		
Test for directional horizontal pleiotropy	0	0	0.816		
**Breast cancer (GWAS) || id:ieu-a-1168**	**Method**	**nsnp**	**β**	**se**	**pval**
Results from two sample MR	MR Egger	9	0.478	0.621	0.467
	Weighted median	9	-0.055	0.107	0.607
	IVW	9	-0.111	0.215	0.605
	Simple mode	9	0.054	0.134	0.699
	Weighted mode	9	-0.081	0.123	0.528
	**Method**	**Q**	**Q_df**	**Q_pval**	
Heterogeneity tests	MR Egger	72.5	7	0	
	IVW	83.1	8	0	
	**egger_intercept**	**se**	**pval**		

Test for directional horizontal pleiotropy	-0.041	0.04	0.346		
**Colorectal cancer || id:ieu-b-4965**	**Method**	**nsnp**	**β**	**se**	**pval**
Results from two sample MR	MR Egger	9	0.001	0.005	0.806
	Weighted median	9	-0.001	0.002	0.839
	IVW	9	-0.002	0.002	0.234
	Simple mode	9	0.000	0.004	0.933
	Weighted mode	9	0.001	0.004	0.887
	**Method**	**Q**	**Q_df**	**Q_pval**	
Heterogeneity tests	MR Egger	4.27	7	0.748	
	IVW	4.78	8	0.781	
	**egger_intercept**	**se**	**pval**		
Test for directional horizontal pleiotropy	0	0	0.5		
**Lung cancer || id:ieu-b-4954**	**Method**	**nsnp**	**β**	**se**	**pval**
Results from two sample MR	MR Egger	9	0.007	0.004	0.116
	Weighted median	9	-0.002	0.002	0.322
	IVW	9	-0.002	0.001	0.251
	Simple mode	9	-0.004	0.003	0.207
	Weighted mode	9	-0.004	0.003	0.308
	**Method**	**Q**	**Q_df**	**Q_pval**	
Heterogeneity tests	MR Egger	4.02	7	0.778	
	IVW	9.67	8	0.289	
	**egger_intercept**	**se**	**pval**		
Test for directional horizontal pleiotropy	-0.001	0	0.049		
**Cancer of skin || id:ukb-d-C_SKIN**	**Method**	**nsnp**	**β**	**se**	**pval**
Results from two sample MR	MR Egger	9	0.007	0.010	0.492
	Weighted median	9	-0.002	0.004	0.631
	IVW	9	-0.001	0.003	0.778
	Simple mode	9	-0.005	0.008	0.500
	Weighted mode	9	-0.004	0.007	0.589
	**Method**	**Q**	**Q_df**	**Q_pval**	
Heterogeneity tests	MR Egger	7.92	7	0.34	
	IVW	8.79	8	0.36	

	egger_intercept	se	pval
Test for directional horizontal pleiotropy	-0.001	0.001	0.41

Table S2. Mendelian Co-localization Analysis for HF

snp-root	analyte	chr	p0sition	gene	location	effect	other_allele	eaf	maf	pval	beta	se	snp
rs2519093	IL-3 Ra	9	136141870	ABO	trans	T	C	0.178	0.178	8.3E-198	-0.86	0.03	rs600038
rs2519093	GP116	9	136141870	ABO	trans	T	C	0.178	0.178	1.4E-155	-0.78	0.03	rs600038
rs2519093	C1GLC	9	136141870	ABO	trans	T	C	0.178	0.178	1.9E-89	0.61	0.03	rs600038
rs635634	VEGF sR2	9	136155000	ABO	trans	T	C	0.18	0.18	4E-80	-0.58	0.03	rs600038
rs507666	IR	9	136149399	ABO	trans	A	G	0.179	0.179	4.8E-80	-0.58	0.03	rs600038
rs2519093	VEGF sR3	9	136141870	ABO	trans	T	C	0.178	0.178	5.9E-80	-0.58	0.03	rs600038
rs2519093	GLCE	9	136141870	ABO	trans	T	C	0.178	0.178	1.1E-78	0.58	0.03	rs600038
rs507666	SELE	9	136149399	ABO	trans	A	G	0.809	0.191	1.01E-52	-0.882	0.057715449	rs600038
rs2519093	P-Selectin	9	136141870	ABO	trans	T	C	0.178	0.178	4.3E-48	-0.46	0.03	rs600038
rs115478735	ISLR2	9	136149711	ABO	trans	T	A	0.178	0.178	1.1E-46	-0.45	0.03	rs600038
rs635634	sICAM-5	9	136155000	ABO	trans	T	C	0.18	0.18	3.4E-40	-0.42	0.03	rs600038
rs635634	Met	9	136155000	ABO	trans	T	C	0.18	0.18	3.2E-33	-0.38	0.03	rs600038
rs8176643	TLR4:MD-2 complex	9	136149709	ABO	trans	A	AC	0.171	0.171	4E-24	-0.33	0.03	rs600038
rs115478735	TPST2	9	136149711	ABO	trans	T	A	0.178	0.178	7.9E-24	0.32	0.03	rs600038
rs2519093	B3GN2	9	136141870	ABO	trans	T	C	0.178	0.178	4.2E-23	0.31	0.03	rs600038
rs635634	LIF sR	9	136155000	ABO	trans	T	C	0.18	0.18	6E-21	-0.3	0.03	rs600038
rs2519093	FAM3B	9	136141870	ABO	trans	T	C	0.178	0.178	3.1E-20	0.29	0.03	rs600038
rs2519093	THSD1	9	136141870	ABO	trans	T	C	0.178	0.178	2.7E-19	-0.29	0.03	rs600038
rs635634	sICAM-5	9	136155000	ABO	trans	T	C	0.18	0.18	6.9E-18	-0.28	0.03	rs600038
rs507666	sICAM-1	9	136149399	ABO	trans	A	G	0.179	0.179	1.6E-17	-0.27	0.03	rs600038
rs5877291	FA20B	9	136138765	ABO	trans	G	GCGCCCAC	0.178	0.17	7.1E-17	0.27	0.03	rs600038

26							CACTA		8				
rs587729126	B4GT1	9	136138765	ABO	trans	G	GCGCCCACCACTA	0.178	0.178	3.5E-14	0.24	0.03	rs600038
rs587729126	sICAM-2	9	136138765	ABO	trans	G	GCGCCCACCACTA	0.178	0.178	6.6E-14	-0.24	0.03	rs600038
rs635634	Endoglin	9	136155000	ABO	trans	T	C	0.18	0.18	1.3E-13	-0.24	0.03	rs600038
rs635634	gp130. soluble	9	136155000	ABO	trans	T	C	0.18	0.18	5.2E-13	-0.23	0.03	rs600038
rs587729126	DPEP2	9	136138765	ABO	trans	G	GCGCCCACCACTA	0.178	0.178	1.5E-12	-0.23	0.03	rs600038
rs8176643	MA1A2	9	136149709	ABO	trans	A	AC	0.171	0.171	6.2E-12	0.23	0.03	rs600038
rs635634	IGF-I sR	9	136155000	ABO	trans	T	C	0.18	0.18	1.1E-11	-0.22	0.03	rs600038
rs2519093	ACE	9	136141870	ABO	trans	T	C	0.928	0.072	0.000000019	-0.352	0.062623776	rs600038

Table S3. SMR analysis results of HF

Gene	topSNP	b_GWAS	se_GWAS	p_GWAS	b_eQTL	se_eQTL	p_eQTL	b_SMR	p_SMR	p_HEIDI
ABO	rs600038	-0.0569	0.0096	3.67697E-09	-0.408008	0.0470884	4.52614E-18	0.139458	0.0285072	9.97981E-07
SURF1	rs600038	-0.0569	0.0096	3.67697E-09	0.287458	0.0499886	8.89963E-09	-0.197942	0.0479601	3.67172E-05
